# Predictors of risky sexual behaviour among pre-college students in Adama Town, Ethiopia

**DOI:** 10.11604/pamj.2019.33.135.18068

**Published:** 2019-06-24

**Authors:** Geremew Werkeshe Wana, Oyedunni Arulogun, Adebola Roberts, Abraham Sahilemichael Kebede

**Affiliations:** 1 Pan Africa University Life and Earth Sciences Institute (PAULESI), University of Ibadan, Ibadan, Nigeria; 2 Department of Health Promotion and Education, Faculty of Public Health, College of Medicine, University of Ibadan, Ibadan, Nigeria; 3 Department of Obstetrics and Gynaecology, Faculty of Clinical Sciences, College of Medicine, University of Ibadan, Ibadan, Nigeria

**Keywords:** Risky sexual behaviour, attitude, social media usage, sexual orientation, reproductive health

## Abstract

**Introduction:**

The rate of sexually transmitted infection's, including HIV has increased in recent years in Ethiopia. Many adolescents and young people still do not protect themselves against unintended pregnancies and STIs. Therefore, this study was conducted to assess the predictors of risky sexual behavior among pre-college students in Adama Town, Ethiopia.

**Methods:**

School based cross-sectional study was employed. In this study 364 students were recruited from all pre-college schools in Adama town, Ethiopia. Bivariate and multivariate logistic regression analysis were used to examine the relationship between the outcome variables and independent variables.

**Results:**

The mean age at sexual debut was 16.1 years (± 2.72SD). Social media usage for sexual activity and having multiple sexual partners were observed among students. About 7% of students used social media for watching pornography. The odds of risky sexual behaviour were higher among social media users compared to the nonusers AOR = 1.23 (95% CI 1.13,3.12). Risky sexual behaviour was almost 4 times more likely among night club goers AOR = 4.294 (95% CI: 2.033, 9.073). Peer pressure and substance abuse were also a significant predictor for risky sexual behavior AOR = 6.97 (95% CI: 4.24, 9.69).

**Conclusion:**

Social media use, peer pressure, substance abuse, and night club going were found to be significantly associated with risky sexual behaviour among pre-college students. Thus, schools need to establish and strengthen reproductive health clubs to be able to equip students with required skills and knowledge about sexuality. Parents should be aware of the dynamic behavioral change of their children, listen and attend to their needs.

## Introduction

Sexual activities among adolescents have been reported to be increasing worldwide. Several studies in sub-Saharan Africa (SSA) have demonstrated high and increasing premarital sexual activities among adolescents [1]. Risky sexual behaviors, including early sexual debut, unprotected sexual intercourse, and multiple sexual partners, occur in a broader context. The intensity of involvement in such behavior ranges from non-sexual relationship to unprotected sexual intercourse with multiple partners [2]. There are an estimated 24.7 million people living with HIV (Human Immune Virus) in sub-Saharan Africa, nearly 71% of the global total. Ethiopia is among 10 countries which contributes 81% of all people living with HIV in the region. The number of women living with HIV in the region is much higher than that of men. Women account for 58% of the total number of people living with HIV. There are about 2.9 millions of children (aged between 0 and 14) and similarly, 2.9 millions of young people (aged between 15 and 24) are living with HIV in the region as well [3].

The rate of STIs (Sexually Transmitted Infection's), including HIV has increased in recent years in Ethiopia. Many adolescents and young people still do not protect themselves against unintended pregnancies and STIs. A study in Ethiopia showed that the median age at first sex is dropping: for males 16 - 18 years and for females 13 -15 years [4]. Another study in Bahir Dar also showed that there is an association between watching pornographic videos, attending night clubs, khat chewing and taking alcohol frequently with having unsafe sex and having multiple sexual partners [5]. The reason given for the rapid lowering of the early sexual debut is associated with a negative aspect of globalization, lack of open discussion with parents about sexual issues, peer pressure, use of different substances (like khat, cigarette, alcoholic drinks) and unplanned money [6]. Studies in different part of Ethiopia also showed condom use is very low: study in Jiga showed that only 8 (16.7%) of the respondents who had started having sex used condom during the first sex, while 40 (83.3%) did not use condom at first sex [7]; study in Dessie also showed that there were only 21 percent of sexually experienced participants who used condom consistently. The reasons reported for not using a condom consistently were: lack of information (42%); religious case (18.8%); fear of side effect (18.2%); a cultural taboo (13.1%); and almost 8% trusted their sexual partner and it was also reported that about 60% of pregnancies are unwanted or unintended in Ethiopia [1,7]. Thus, this study was designed to highlight the attitudes and predictors of risky sexual behaviors among pre-college students in Adama town, Ethiopia.

## Methods

### Study design and study area

A cross sectional study design was employed. This study was conducted in Adama town, one of the commercial cities found in Oromia State, Ethiopia. The city is found 100km in the south-east of the capital Addis Ababa. The recent national census revealed that the total population of the town is expected to be half a million [8].

### Sampling

Sample size for the study was determined by using single population proportion formula. To calculate the sample size; 71% prevalence of risky sexual behaviour among sexually active students from a study conducted in Tis Abay, Amhara region were considered [9]. 95% confidence interval, 5% margin of error and population correction formula were used to estimate the final sample size. Finally, 15% non-response rate was included to get the final sample size 364. Samples were proportionally allocated based on the size of students in each school. Hence, stratified sampling method was used to draw samples from pre-college schools found in the town. This study was conducted between April and May, 2017.

### Measurements

#### Dependent variable

In order to classify whether the study participant (students) were risky or not, three criteria were used. Exhibiting one or more of the following characteristics were used to categorize individuals to risky or not risky. The criteria were; multiple sexual partner: a student who have a sexual relationship with more than 2 partners have been considered as risk and if the participant had only one, he was considered not risky; unprotected sex: respondents who always used condom in every sexual activity in the past 13 months were considered as non-risky and on the other part respondents who used condoms irregularly or not at all were considered as risky; Early sexual debut: respondents with sexual initiation of less than 15 years of age were considered as risky and other wise non risky.

#### Data Collection Tools

A self-administered questionnaire adopted and modified from literatures and using the standard EDHS questionnaire to collect the data. The questionnaire was first prepared in English then translated to the local language, Amharic, by language expert. Prior to data collection, every section of the questionnaire was pre-tested on 5% of the sample size on school found outside the Adama town. Training were given to the data collectors before the commencement of the data collection. On spot monitoring and supervision were done.

#### Analysis

Epi-data version 4.2 software was used to enter the data. Data cleaned, coded and exported to STATA version 13 for further analysis. Descriptive statistics, frequency tables, percentages, mean median and proportion were used to summarize the collected data. The bivariate and multivariate analysis were used to identify predictors of risky sexual behaviour and to adjust for possible confounders, at a p-value less than 0.05.

#### Ethical approval

Ethical approval letter to conduct this study were obtained from Institute for Advanced Medical Research and Training (IAMRAT), College of Medicine, University of Ibadan, Ibadan, Nigeria and Oromia Regional Health Bureau Ethical Committee. Prior to the data collection, permission letter was granted from Oromia Regional State Education Bureau, Adama Woreda Educational Office and from every pre-college schools included in the study. Informed consent was obtained from all individual participants included in the study.

## Results

### Socio-demographic characteristics of the students

Respondents were made up of 56.4% males and 43.6% females. Majority (56.6%) of them were aged below 18 and 96% of them were singles. Around 82.9% of the respondents were either living or came from the urban areas. More than 50% of the respondents were Orthodox in religion (Table 1).

**Table 1:** Socio-demographic characteristics of respondents

Variables	Frequency N (346)	Percentage
**Sex**		
Male	195	56.4
Female	151	43.6
**Age**		
<18	196	56.6
>18	150	43.4
**Educational status**		
Grade 11^th^	137	39.6
Grade 12^th^	209	60.4
**Educational performance**		
Poor	14	4.0
Satisfactory	24	6.9
Good	217	62.7
Very good	91	26.3
**Residence**		
Urban	253	73.1
Rural	93	26.9
**Ethnicity**		
Amhara	131	37.9
Tigre	21	6.1
Oromo	164	47.4
Others	30	8.7
**Religion**		
Orthodox	197	56.9
Islam	54	15.6
Protestant	85	24.6
Catholic	2	6
Others	8	2.3
**Marital status**		
Single	332	96.0
Married	8	2.3
Other	6	1.7

### Social media usage and its influence among pre-college students in Adama town

More than 89% of the study participants were social media users. Facebook, WhatsApp and Viber were common social media platforms the students use, that almost 90%, 37% and 34% of the students used them respectively. Forty percent of the students used social media every day. Regarding the purpose of using those media, 69.7% use them for interaction purpose, 34.4% for entertainment, 36% to see news updates and 20.5% to watch movies. Seven percent (7.3%) of the students were using social media to watch porn movies. More than 45% of the respondents start viewing pornographic materials between the age of 15 and 19 years (Table 2)

**Table 2:** Social media usage and its influence on the respondent’s behaviour

Variables	Frequency	Percentage
**Social media use**		
Yes	311	89.9
No	35	10.1
Total	346	100.00
**Type of social media used**		
Facebook	283	90.9
Viber	107	34.4
WhatsApp	117	37.6
Youtube	104	33.4
Instagram	69	22.1
Total	311	100.00
**Social media usage frequency**		
Many times a day	69	22.2
Every day	127	40.8
Every week	44	14.4
Once in a while	71	22.8
Total	311	100.00
**Purpose of the use (multiple answers)**		
For interacting with friends	217	69.7
For entertainment	107	34.4
For the News updates	112	36
To watch a movie	64	20.5
To watch pornography	23	7.3
Total	311	100.00
**Age at first viewing of porn videos or pictures**		
10-14	9	7.3
15-19	56	45.5
20-24	56	45.5
24+	2	1.6
Total	123	100.00

### Individual factors influencing risky sexual behaviour

13.6% students mentioned peer pressure as a pushing factor to engage in risky sexual activities. Out of those reported peer pressure, 28.6% went to the night clubs for the first time with their peers. On the other hand, 46.9% students reported that they were going out to drink alcohol every week due to peer influence that almost 8% ended up with casual sex. Almost half (46.9 %) of students were unable to resist peer influences because of the fear to be called nerd among their friends and 38.7% were afraid of isolation. Almost half (49.6%) drank excessively and 26% had casual sex while they were at the club. 42.2% students got drunk in the last three months, while 11.3% smoked cigarette. More than 10% and 6.4% of the study participants chewed khat and smoked weed/shisha respectively. In relation to the frequency of the intake, 36.1% of them took alcohol a couple of times in the week. Thirty-four percent of them chewed khat every week and 27.2% took shisha/hashish every day.

### Knowledge about STI's and HIV-AIDS

Almost all (94.8%) respondents had heard about STI's. On STI mode of transmission 94.5% responded STI is transmitted due to unsafe sex, 80.5% by facing the sun during urination, 70.1% urinating on the hot place, 54.9% vertical transmissions and 40.8% through blood transfusions. Similarly, more than 90% student’s knowledge on HIV mode of transmission was observed. 7.5% of the students believe HIV can be contracted by shaking hands and dining with an infected person. Abstinence (81.2%), limiting to one partner (35%) and using a condom (38.9%) were among the preventive methods chosen by students. About 95.7% students believed STIs are preventable diseases. Using condom during sexual intercourse (64.9%), douching (56.5%), avoiding casual partner (52.9%), abstinence (50.8%) and avoiding sex with commercial sex workers (64.6%) were mentioned as strategies to prevent STI's by the participant students. While 71.9% of them said STI can be prevented by using contraceptive pills.

### Sexual behaviour of respondents

**Figure 1:**
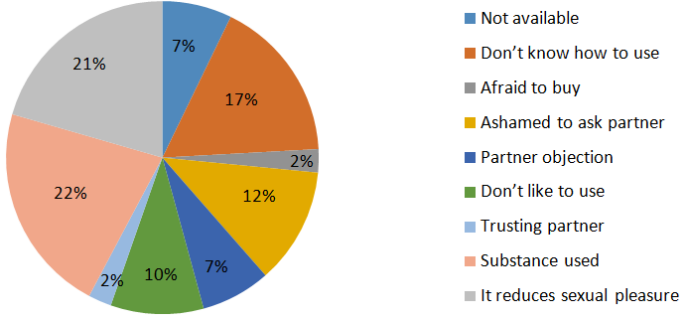
Reasons for not using condom among pre-college students in Adama town, Ethiopia

One third (31.8%) of respondents engaged in the sexual activities. Over 98% of them started sex below the age of 18. Thirty-five percent, 31%, and 29% of the students engaged in masturbation, anal, oral types of sex. Most of them (36.5%) had their first sexual intercourse with their steady boy/girlfriend, 34.5% had it with casual sexual partners. About 12.7% reported their first sexual intercourse was with family members and 10% of them with their teachers. Over 90.9% of students said that their first sexual partners' age varied between five years and less. Non-condom use was reported on the 47.3% of student's first sex involvement. As at the time of the data collection, 30% students had more than two partners and 17.3% of these had over three partners. Thirty-two percent of the students had engaged in sex in return for money; transactional sex. On the other hand, 37.3% students had sex after alcohol intoxication, 25.4% had sex after chewing khat while the rest 26.9% used hashish before sex (Table 3). Consistent condom use during every sexual intercourse among study participants were 33.3%. Excessive alcohol drinking (22%), afraid to buy (21%), not knowing how to use it (16.9%), and mis-conceptions like condom reduces sexual pleasure (20.5%) were listed as the reason for the inconsistent condom use by the students (Figure 1).

**Table 3:** Sexual behaviour of respondents

Variables/Categories	Frequency	Percentage
**Ever had sexual experience(n=346)**		
Yes	110	31.8
No	236	67.6
**Age at first sexual experience(n=110)**		
10-14	8	7.3
15-18	100	90.9
18+	2	1.8
**Type of sex exercised (n=278)**		
Coitus	10	3.6
Anal	87	31.3
Oral	82	29.5
Masturbation	99	35.6
**With who was the first intercourse(n=110)**		
Steady boy/girl friend	40	36.5
Casual boy/girl friend	38	34.5
Husband/ wife	4	3.6
Family member	14	12.7
Teacher	11	10
Commercial sex workers	3	2.7
**How many years older was your partner at first intercourse(n=110)**		
<5years	100	90.9
5-10years	10	9.1
**Number of sexual partners ever (n=110)**		
>3	33	30
2	19	17.3
1	58	52.7
**Type of sex exercised (n=163)**		
Coitus	12	7.4
Anal	43	26.4
Oral	47	28.8
Masturbation	61	37.4
**Condom use at first contact(n=110)**		
Yes	58	52.7
No	52	47.3
**Frequency of condom use (n=67)**		
Always	12	17.9
Sometimes	24	35.8
Not at all	31	46.3
**Transactional sex experience in the last 3 months (n=67)**		
No	45	67.2
Yes	22	32.8

### Factors associated with risky sexual behaviour

Social media usage for sexual activity, Age of the respondent, educational status, peer pressure, night club going, use of addictive substances such khat, cigarette and alcohol were found to have significant association with risky sexual behaviour. After considering the existing confounders in bivariate analysis, those variables found to be significant was retained for multivariate analysis to see individual effect using P-value < 0.05. The odds risky sexual behaviour was higher among social media users compared to social media nonusers AOR: 1.3(95% CI: 1.23, 3.12). The odds of risky sexual behaviour were 4 times more among night club goers compared to those non goers (AOR: 4.294, 95%CI: 2.033, 9.073). Peer pressure (AOR: 6.97, 95% CI: 4.24, 9.69), smoking cigarette (AOR: 0.505, 95% CI: 0.004, 0.649) and drinking alcohol (AOR: 0.461, 95% CI: 0.232, 0.916) found to be significantly determine the risky sexual pattern among the pre-college students in Adama town (Table 4).

**Table 4:** Bivariate and multivariate logistic regression analysis of socio-demographic and economic factors on risky sexual behaviour

Characteristics	Risky sexual behavior		COR (95%CI)	AOR (95%CI)
	Yes	No		
**Age**				
<18	51	145	0.53 (0.33, 0.83)*	
>18	60	90	ref	
**Residence**				
Urban	79	174	0.86 (0.52, 1.43)	
Rural	32	61	ref	
**Religious attendance**				
Yes	97	190	1.64 (0.86, 3.14)	
No	14	45	ref	
**Discussion with parents about RH issues**				
Yes	53	85	1.61 (1.02, 2.55)	
No	58	150	ref	
**Pressure from peer to have sex**				
Yes	42	5	28.84 (10.98,75.79)*	6.97 (4.24,9.69)*
No	67	230	ref	
**Chewing khat in the last three months**				
Yes	44	6	0.04 (0.02, 0.10)*	0.61 (0.14, 2.64)
No	67	229	ref	
**Smoking shisha in the last three months**				
Yes	21	1	2.464 (0.99, 6.12)	0.42 (0.03, 5.95)
No	90	234	ref	
**Age at first sexual experience (years)**				
Below 15	2	1		
Above 15	109	0		
**Night club visit for the last 3 months**				
Yes	57	24	9.637 (5.48, 16.96)*	4.29 (2.03, 9.07)*
No	52	211	ref	
**Smoked cigarette in the last three months**				
Yes	38	1	0.008 (0.01, 0.06)*	0.51 (0.04, 0.65)*
No	73	234	ref	
**Drinking alcohol in the last three months**				
Yes	80	66	0.14 (0.09, 0.24)*	0.46 (0.23, 0.92)*
No	29	168	ref	
**Social media use**				
Yes	105	206	2.46 (1.99, 6.12)*	1.23 (1.13,3.12)*
No	6	29	ref	
**RH club at your school**				
Yes	60	152	0.64 (0.41, 1.02)	
No	51	83	ref	
**Age at first viewing of porn videos or pictures**				
10-14	6	3	ref	
15-19	34	22	0.77 (0.18, 3.42)	
20-24	34	22	0.77 (0.17, 3.42)	
**Heard of STI before**				
Yes	107	221	0.59 (0.19, 1.84)	
No	4	14		

* = p-value<0.05 (significant), AOR: Adjusted Odds Ratio, COR: Crude Odds Ratio, RH: reproductive health

## Discussion

This study was aimed to assess the predictors of risky sexual behavior among pre-college students in Adama Town, Ethiopia. This study found out that, social media use, substance abuse, peer pressure and night club going were associate with risky sexual behaviour. In this study, among those who had sex over 98.2% of them started sex before the age of 18 which makes them much riskier than that of those who started sex above the age of 18 years of age. This finding was in line with the study from the South East of Ethiopia, Medawelabu University which revealed that 42.7% of students had admitted to premarital sexual intercourse with the average age of initiating sexual intercourses was 18.4 ± 2.14 years. The higher proportion in this study might be due to the fact that Adama is more urbanized than Medawelabu [10,11]. Almost one-third (31.2%) of respondents had a sexual relationship with non-regular partners in the previous one year and of those, 58.5% had sex with two or more different partners [12]. Comparably, a study conducted in Jiga High school, Northern Ethiopia reported that out of sexually active respondents, 66.4% had one sexual partner, and 33.6% had two or more sexual partners [7,11]. This could also be due to students' favorable attitude towards having multiple concurrent sexual partners in preparatory level students in the current study. Furthermore, it might also be due to the age group difference and socio-demographic factors variation. The current study reported that religiosity was the very important factor in determining risky sexual behavior with 82.9%. Likewise, a study from Delta state, Nigeria showed that there were about 43% of the variation of risky sexual behavior among adolescents was accounted for by the independent variables, the most potent being religiosity [13]. The figure might be higher due to the fact that Ethiopia is a more traditional nation in terms of attending religious services at least once in a week which is especially very common among Ethiopian orthodox coptic church.

A study among gays in America identified socio-contextual factors as a key factor for engaging in the risky sexual behavior. This study investigated factors associated with sexual behavior that confers the greatest risk for HIV transmission (i.e., unprotected anal intercourse; UAI) [14]. In this study also there were 26.4% and 28.8% of students who practiced anal and oral sex in the last three months respectively. Regarding the attitude of students towards homosexuality, about 43.9% of the students strongly supported the statement that saying “It's fine to have oral and anal sex?”The transformation of the attitude of students towards new types of sex (anal and oral) was probably due to the influence of globalization and the universal acceptability of homosexuality in international conventions and becoming part of educational curriculums [15]. Out of 48 respondents who had sex, only 8 (16.7%) used a condom, and the rest 83.3% did not use a condom at first sex in a study conducted in Bahir Dar, Ethiopia [16]. Relatively, this study has also come up with the report that 31.8% of students were engaged in sexual intercourse though 47.3% of students didn4t use a condom in their first sex. This could be due to the nature of the study which the former was correlational whereas this study was cross-sectional. Other possible explanation for the difference of result could be the awareness and skills of the students about condom usage might be higher in Adama. Substance users were about 2.5 times more likely to be involved in risky sexual behavior compared to nonusers and those drinking alcohol daily were 3.5 times more likely to be involved in risky sexual behaviour [17]. Moreover, in other study found that the combination of alcohol use, the freedom associated with living away from home and the social expectations of a risky student sexual lifestyle seem to play a central role in shaping the risky sexual lifestyle of university students in the UK [18]. Likewise, in this study students' knowledge about the ill effects of drugs were also high but many were involved in it. Those who smoked cigarette were twice more likely to engage in risky sexual behavior than those who didn't smoke.

Peer pressure is found to be the most important factor associated with risky sexual behavior among school adolescents in a study conducted in Addis Ababa. The finding further explained that risky sexual behavior was significantly and very strongly associated with the perception of peers' involvement in sexual intercourse [19]. This could be mainly due to the limited organizations which work with adolescents and, very few youth-friendly clinics found in the Adama as compared with Addis. As a result, students easily influenced by their peers to engage in risky sexual behaviors [20]. This study also came up with the finding that 89.9% of students use social media. Out of which, Facebook use was 90.9%. Among those using social media, 7.3% students reported that they had used social media to watch porn movies. Social media such as Facebook, Instagram, YouTube and others have been identified as a factor that alters adolescent's perception and influences the norm that predisposes them to engage in RSB. A comparative study in the USA stated that those who viewed sexually suggestive Facebook photos had a higher chance of having unprotected sexual intercourse and sex with strangers [21]. This study was in line with other studies done in USA and Europe [22,23]. The odds of risky sexual behavior was 4 times higher among students going to night clubs. In line to this finding, a school-based studies in Amhara, Ethiopia found 4 times higher risky sexual behaviours among night club goers [11]. This might be due to the existing high peer pressure and the higher availability of night clubs the towns.

***Limitation of the study:*** given the nature of the study design, we can't establish a temporal relationship. This study was done in an urban set up it should have been great if a comparison group from rural residents were included.

## Conclusion

Social media usage, drinking alcohol, smoking cigarette, peer pressure and night club going were found to have a strong association with risky sexual behavior. Knowledge of contraceptive, STI including HIV was high though the practice was not in line with the knowledge. This shows the rampant attitudinal problem and skill gaps in this age group. Youth-friendly clinics where students can get those RH services were not available and students didn't know where to go when they seek for any of these RH services. Even if they went to those public health institutions, they had not treated the way they need to be treated as the service was not designed for youth but for the adults. Further and rigorous to establish the cause effect relationship between social media use and risky sexual behaviour is needed to document and make a recommendation.

### What is known about this topic

- Adolescents are prone to risky sexual behaviours;
- Peer pressure are the common mentioned predictors of risky sexual behaviour in several studies;
- Early sexual initiation has been documented.

### What this study adds

- The impact of social media usage among adolescents in Ethiopia were unstudied;
- Purpose of social media usage among adolescents in precollege have been identified;
- Indicated the need for further ethnographic studies on utilization of social media for sexuality purposes.

## Competing interests

The authors declare no competing interests.
